# Coptisine enhances the sensitivity of chemoresistant breast cancer cells by inhibiting the function and expression of ABC transporters

**DOI:** 10.3389/fphar.2024.1472458

**Published:** 2024-12-03

**Authors:** Safaa Yehia Eid

**Affiliations:** Department of Biochemistry, Faculty of Medicine, Umm Al-Qura University, Makkah, Saudi Arabia

**Keywords:** coptisine, multidrug resistance, ABC-transporters, breast cancer, P-glycoprotein

## Abstract

**Background:**

Multidrug resistance (MDR), mainly caused by ATP-binding cassette transporters (ABCTs) efflux, makes it difficult for many anticancer drugs to treat breast cancer (BC). Phytochemicals can reverse cancer’s MDR by modifying ABC transporter expression and function, as well as working synergistically with anticancer drugs to target other molecules. The reversal effect of the isoquinoline alkaloid coptisine (COP) was assessed on four breast cell lines; Two sensitive MCF-7 cell lines with positive estrogen, androgen, progesterone, and glucocorticoid receptors, as well as MDB-MB-231 cells with negative estrogen, progesterone, and HER2 receptors, and two doxorubicin-resistant cell lines, MCF-7/ADR and MDB-MB-231/ADR.

**Methods:**

The cytotoxicity of COP and its ability to improve doxorubicin (DOX) cytotoxicity were assessed using the MTT assay. The effectiveness of COP in reversing DOX resistance was evaluated by calculating resistance ratio (RR) values, combination index (CI), and isobologram (IB). The inhibitory effect of COP on ABCT efflux function in comparison to verapamil (VER) was evaluated by measuring the cellular accumulation of Rho123 using flow cytometry. The impact of COP, either alone or in combination with DOX, on the gene expression of ABCTs (P-gp/MDR1, BCRP, and MRP1) of investigated cell lines was assessed by RT-PCR.

**Results:**

The COP showed modest cytotoxicity on the examined cell lines. In MCF-7/ADR and MDA-MB-231/ADR cells, COP (31 μM) enhanced DOX cytotoxicity with CI (0.77 and 0.75), RR (2.58 and 3.33), and IB suggesting synergism. COP significantly inhibits ABCT function in resistant BC cell lines, increases Rho123 accumulation, and decreases efflux more than VER; 2.1 and 1.2-fold, respectively. The combination of COP and DOX had a strong inhibitory effect on ABCT function (3.1 and 3.9 times VER, P< 0.001) and downregulated the genes and protein expression of ABCT.

**Conclusion:**

COP reversed ABCT-mediated multidrug resistance *in vitro*, indicating its potential as a multidrug resistance-reversing agent in cancer chemotherapy.

## 1 Introductions

Breast cancer (BC) is a malignancy commonly detected in women, characterized by a significant global mortality rate. The global incidence of breast cancer reached 2.3 million cases in 2022 (Ferlay J et al., 2024). As the most often diagnosed cancer in females, it represents around 23% of all cancer cases (Trapani et al., 2022). The molecular differences observed in breast tumors necessitate the use of multiple drugs to treat this illness. In addition to surgery, chemotherapy, and radiotherapy, which form the foundation of breast cancer treatment, targeted therapy using drugs that specifically target molecular receptors appears to be a promising approach in many conditions (Dodelzon et al., 2021). One example of this is endocrine therapy, which uses the selective estrogen receptor modulator tamoxifen to target and restrict the proliferation of breast cancer cells that are positive for the estrogen receptor (ER), ultimately leading to their cell death by apoptosis (Li et al., 2017). In contrast, the triple-negative breast cancer (TNBC) subtype has the highest probability of recurrence and the most unfavorable prognosis for survival (Yin et al., 2020).

Anthracycline drugs are frequently used as chemotherapeutic agents for malignant breast cancer. People who are resistant to endocrine therapy or have metastatic cancer extensively use doxorubicin (DOX) a commonly prescribed anthracycline drug, to treat their breast cancer (Shen and Liu, 2023). Although the specific mechanisms underlying its chemotherapeutic efficacy are not fully understood, DOX primarily promotes apoptotic cell death in cancer cells (Kciuk et al., 2023). When administered as a solitary treatment, Dox generally achieves response rates ranging from 40% to 60%, with the possibility of reaching as high as 80% (Rahman et al., 1999). Despite the effectiveness of initial cancer treatment, the emergence of DOX (doxorubicin) resistance poses a significant challenge in medical practice and significantly contributes to treatment failure (Rahman et al., 1999). Researchers have proposed various mechanisms to develop multidrug resistance (MDR) to DOX in breast cancer. It could be an intrinsic or acquired DOX resistance. Secondary resistance develops when breast cancer cells that were previously responsive to DOX become resistant during treatment (Sadida et al., 2024).

MDR represents acquired resistance to numerous anticancer drugs with different structures or mechanisms. Furthermore, MDR cells can gain tolerance to new toxic substances. Several biological pathways can produce multidrug resistance. The most common mechanism is transmembrane protein overexpression, which causes typical MDR (Assaraf et al., 2019). These proteins eliminate chemicals from the cell in one direction, lowering the concentrations of cytotoxic agents below the effective level. The most abundantly generated transport proteins in MDR tumor cells are ATP-binding cassette transporters (ABCTs). ATP hydrolysis powers these proteins’ active transport of chemotherapeutic drugs against the concentration gradient (Teodori et al., 2022). Overexpression of these proteins increases chemotherapy efflux outside cancer cells and therapy failure, indicating MDR (Nobili et al., 2020). However, these proteins are abundant in different healthy tissues and perform various physiological and pharmacological functions (El-Readi et al., 2021). Their presence protects tissues from xenobiotics while also affecting absorption, distribution, metabolism, excretion, and toxicity (ADMET) as well as drug bioavailability. P-glycoprotein (P-gp/ABCB1/MDR1), multidrug resistance protein 1 (MRP1/ABCC1), and breast cancer-resistant protein (BCRP/ABCG2/MXR) are the most important members of this family (Ashique et al., 2024).

As a result, the effectiveness of existing therapeutic methods in BC treatment is insufficient, creating a significant need for more research in this area. Regardless of some unsuccessful outcomes, natural products are still being considered as a potential solution to the difficulties that may arise with MDR in breast cancer (Malla and Kiran, 2022). Reports indicate that they can reverse its MDR by modulating ABCT, inhibiting cell cycle progression, inducing cell death, reducing recurrence, and stopping metastasis while causing few side effects (El-Readi et al., 2024; Wink et al., 2012; El-Readi et al., 2021).

**FIGURE 1:**
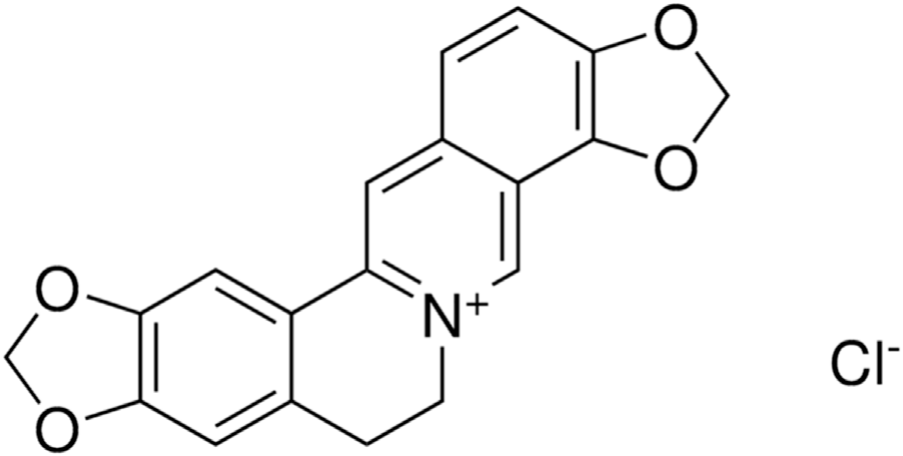
Chemical structure of Coptisine chloride.

Coptisine (COP) is one of the major secondary metabolites present in *Rhizoma coptidis*. It is a natural protoberberine alkaloid commonly found in the Ranunculaceae and Papaveraceae plant families (Figure 1) (Cao et al., 2012).

Different experimental sets have examined COP’s *in vitro* anti-cancer effects in various cancer cell lines, targeting apoptosis and cell cycle arrest. COP (0–25 μM, 24 h) reduced HCT116 cell viability, adhesion, and migration (Cao et al., 2018). COP inhibits apoptosis by modulating ROS, Bcl-2, Bid, Bax, cytochrome c, Apaf-a, AIF, XIAP, caspase-3, and caspase-9 (Zhang et al., 2018). COP (0–28.11 μM, 24 h; 0–75 μM, 48 h) inhibited HCT-116 cells in G1 phase and reduced expression of key genes like CDK4, CDK2, cyclin E, and cyclin C during the G1/S (Han et al., 2018). COP showed the same anticancer effects on breast cancer cells (Li et al., 2014), non-small cell lung cancer cells (Rao et al., 2017), liver cancer cells (Zhou et al., 2018), and pancreatic cancer cells (Zhang et al., 2019). COP (12.5–100 μM for 24 h) reduced HepG2 cell viability and growth by reducing miR-122 expression. Human hepatoma cells showed increased 67kD laminin receptor/cGMP pathway transduction and apoptosis (Zhou et al., 2018). A subsequent study found that COP (0–64 μM, 24 h) reduced MMP-9 mRNA expression in MDA-MB-231 cells by increasing TIMP-1 mRNA, a known MMP inhibitor (Li et al., 2014). COP dose-dependently inhibited PANC-1 cell metastasis, causing G1 cell cycle arrest and S phase reduction (25–150 μM, 48 h) (Zhang et al., 2019). In non-small cell lung cancer cell line A549, COP (12.5–50 μM, 48 h) caused cell cycle arrest at G0/G1 and G2/M phases, as well as effects on G1/S phase and downregulated Cyclins D/E, CDK2/4/6, Cdc2/25C, and p21 (Rao et al., 2017). COP (12.5–50 μM, 48 h) increased pH2AX protein levels, indicating DNA damage, in A594 cells. Upregulation of ROS, caspase-3/9, Bax/Bcl-2, and PARP cleavage caused apoptosis (Han et al., 2019). Large doses (150 mg/kg) of COP have been shown to hinder *in vivo* tumor development and reduce the probability of tumor cell proliferation. Furthermore, it inhibits the cell cycle’s progress in colorectal cancer cells and induces apoptosis (Huang et al., 2017). Previous studies have documented the potential of COP as an anti-cancer, anti-inflammatory, and antibacterial agent because of its ability to modulate the signaling transduction pathways (Wu et al., 2019; Chai et al., 2024). Virtual screenings, molecular docking of binding affinity, and *in silico* toxicity assessments have previously been used to analyze 375 phytochemicals. An additional round of *in vitro* testing was conducted on COP and five other compounds after these *in silico* results (Schäfer et al., 2023). The multidrug-resistant CEM/ADR5000 cells exhibited cross-resistance to COP, the IC_50_ values were 9.6 µM for CCRF-CEM cells and greater than 250 µM for CEM/ADR5000 cells and moderate P-glycoprotein inhibition and markedly enhanced intracellular DOX accumulation (Schäfer et al., 2023). COP derivative (8-oxocoptisine) exhibits substantial P-glycoprotein multidrug resistance inhibition activity, with an ED50 value of 0.018 μg/mL in MES-SA/DX5 cells and 0.0005 μg/mL in HCT15 cells, respectively. On the other hand, our understanding of how COP and chemotherapy interact to alter ABCT resistance and its effects on breast cancer that has developed resistance is incomplete.

Combining COP with DOX, according to our hypothesis, would increase DOX’s efficacy by facilitating its accumulation within resistant breast cancer cells. Combining COP with DOX is an innovative approach that could protect against the dose-limiting toxicity of DOX while simultaneously increasing its efficacy in cancer treatment.

## 2 Materials and methods

### 2.1 Chemicals and reagents

Chemicals with the highest analytical grade include Coptisine chloride (COP; purity 98%), doxorubicin (DOX or Adriamycin; 99%), Verapamil (VER; 99%), tetrazolium salts (MTT), rhodamine123 (Rho123) were provided by Sigma-Aldrich^®^ (Taufkirchen, Germany). The RNeasy Mini Kit, First Strand cDNA Synthesis Kit for PCR (AMV), and SYBR Green I Kit were acquired from Applied Biosystems (Waltham, Massachusetts, United States). The following cell culture supplies were acquired from Gibco: Penicillin-streptomycin, trypsin-EDTA, glutamine, DMEM media, and fetal bovine serum (FBS) (Thermo Fisher Scientific, NY, United States). The phosphate buffer saline (PBS) solution was acquired from Becton Dickinson, located in Fullerton, CA, United States DMSO and ethanol were acquired from VWR^®^ (Darmstadt, Germany). Cell Signaling Technology Inc. of Massachusetts, United States, supplied all of the primary antibodies.

### 2.2 Cell culture

This study uses four types of breast cancer cells: MCF-7 and MDA-MB-231, which came from the American Type Culture Collection (ATCC, United States); and MCF-7/ADR and MDA-MB-231/ADR cells, which were made by treating them with DOX and growing them in a medium that contained 1.8 µM of DOX to keep their resistance to DOX (El-Readi et al., 2019; Zhang et al., 2015; Zhao et al., 2016). The cell lines were cultured in complete DMEM media with supplemented antibiotics and FBS. The cells were maintained at a temperature of 37°C, with a humidity level of 100% and a CO_2_ concentration of 5%. Regular testing for *mycoplasma* contamination was performed. The cells were collected from fully grown cultures by using a trypsin-EDTA solution. PBS was used to wash the cells. The resistance cell lines were cultured in a medium containing 1.8 µM of DOX to preserve their resistance to doxorubicin. Experiments were conducted using cells that were cultivated for 7–10 days without the presence of any drugs.

### 2.3 Cell viability assay

The MTT assay, as previously published (Carmichael et al., 1987) was used to evaluate the cytotoxic effects of COP and DOX on MCF-7, MCF-7/ADR, MDA-MB-231, and MDA-MB-231/ADR cells. Cells that were growing at an exponential rate were harvested using a solution containing 0.25% Trypsin-EDTA. These cells (2 × 10^3^ cells/well of 96-well plate) were incubated for 24 h. DOX (0.2–184 μM) and COP (0.3–1,561 μM) treated cells for 24 h. Subsequently, the cells were exposed to a 0.5 MTT solution for a duration of 3 h, then DMSO was added to dissolve the formazan crystal, and color density was measured using a SpectraMax II ELISA reader at a wavelength of 570 nm.

### 2.4 Determine synergistic interactions of DOX + COP combination

Resistant cell lines (2 × 10^3^ cells/well) for each cell line and cultivated for 24 h. The cells were treated with DOX 0.2–184 μM plus 31 μM of COP for 24 h and an MTT assay was placed as mentioned above. To evaluate the synergistic interaction between COP and DOX resistance ratio (RR), dose reduction index (DRI), combination index (CI), and isobologram (IB) were applied (Chou, 2006; Chou and Talalay, 1984; Bliss, 1939).

$$\text{DRIcop}=\frac{{\text{IC}}_{50,\text{COP}}}{C_{\text{COP}}}$$

$$\text{DRIdox}=\frac{{\text{IC}}_{50,\text{dox}}}{C_{\text{dox}}}$$

Where, IC50cop and IC50dox are the IC50 of COP and DOX alone and Ccop and Cdox are the concentrations of each drug in combination, respectively.

$$\text{RR}=\frac{{\text{IC}}_{50}\;\text{of}\;\text{resistant}\;\text{cell}\;\text{line}\;}{{\text{IC}}_{50}\;\text{of}\;\text{parental}\;\text{cell}\;\text{line}\;}$$

$$\text{CI}=\frac{C_{\text{DOX},50}}{{\text{IC}}_{50,\text{DOX}}}+\frac{C_{\text{COP}}}{{\text{IC}}_{50,\text{COP}}}$$

Where C_DOX,50=_ IC_50_ DOX in combination with the fixed concentration of COP C_COP_. IC_50, DOX=_ IC_50_ for DOX and IC_50, COP_ = IC_50_ of COP. Synergistic (CI < 1) was determined (Chou, 2010; Chou and Talalay, 1984).

Isobolgram is widely recognized as a valuable tool in multiple medical fields. It provides valuable insights into drug interactions, aids in drug development and combination therapy strategies, and helps optimize treatment regimens. The cytotoxicity results are displayed on a graph, exhibiting the IC_50_ values of DOX on the *x*-axis and COP on the *y*-axis. A line was formed by connecting points that represented the combined effects, resulting in the creation of the isobologram. The isobologram provides valuable insights into drug interaction: The isobologram demonstrates a linear relationship, suggesting that the collective impact is simply the accumulation of the individual effects. Synergistic: The isobologram has a downward curve, indicating that the combined influence is stronger than the mere summation of the separate effects. antagonist: The isobologram curves upward, indicating that the combined effect is lower than the sum of the individual effects.

### 2.5 FACS flow cytometry

The FACSCalibur™, a fluorescence-activated cell sorter manufactured by Becton-Dickinson, San Jose, CA, was used to quantify the fluorescence intensity of accumulated Rho123 inside individual cells. This was achieved by utilizing an ultraviolet argon laser with an excitation wavelength of 488 nm and emission wavelengths of 530/30 nm and 570/30 nm, which were filtered using band-pass filters. The analysis involved the counts of 10,000 cells/sample using forward and side light-scatter plots, specifically focusing on individual viable cells. The log fluorescence was represented as a histogram using a single parameter. A modified MDR efflux pump inhibition experiment was conducted on live-resistant breast cancer cells using a flow cytometer (Ashour et al., 2014). To achieve efflux, the cells were first incubated with Rho123 at a concentration of 10 μg/mL for a duration of 2 h at 37°C. Subsequently, the cells were subjected to treatment with DOX, COP, VER as positive control, and DOX + COP for a duration of 2 h (El-Readi et al., 2019). To normalize the relative fluorescence intensity (inhibitory efficiency) of treated cells, the percentage of VER and negative untreated controls was used as a standard.

$$\text{Inhibitory efficiency}=\frac{\left({\text{RFU}}_{\;\text{Sub}}\;–\;{\text{RFU}}_{\;\text{CT}}\right)}{\left({\text{RFU}}_{\;\text{VER}}\;–\;{\text{RFU}}_{\;\text{CT}}\right)}\;\%$$

RFU_sub_ indicates the fluorescence of the tested substance. RFU_VER_ shows verapamil fluorescence. RFU_CT_ is fluorescence untreated control without any drugs. A drug is considered a good inhibitor of MDR when its maximum inhibition is more than 10%.

### 2.6 RT-PCR

Each cell line was seeded at 1 × 10^6^ cells/well 6-well plates for 24 h. The cells were treated with DOX (1.8 μM), COP (31 μM), and their combination for 48 h. According to the manufacturer’s instructions, RNA was isolated Qiagen RNeasy Mini Kit. RNA was extracted and stored at −80 °C after verifying its quality and purity using a Jenway Genova Nano Spectrophotometer. MDR1, MRP1, and BCRP gene mRNA expressions were evaluated using β2-microglobulin (β2mg) as a housekeeping gene (Table 1) in real-time RT-PCR were obtained from Integrated DNA Technologies, Inc. (Iowa, United States). Reverse transcribed whole RNA into cDNA using a Thermo-Fisher Scientific High-Capacity Reverse transcription kit and manufacturer’s instructions. The cycles (40) of reaction amplification (95 °C for 15 s and 60 °C for 1 min) were used to evaluate the results using the Applied Biosystems 7,500 Fast RT-PCR (Thermo-Fisher Scientific). Sample cycle threshold (Ct) values were compared to the Ct value of negative control (NC). To quantify gene expression, the fold-change 2^−ΔΔCt^ method was used and normalized to β2mg (Eid et al., 2020).

**TABLE 1:** Primers used for real-time qPCR.

Gene	Accession	Forward primer 5′–3′	Reverse primer 5′–3′	Design
*MDR1*/ABCB1	NM_001348946.1 GI: 1149123048	CCC​ATC​ATT​GCA​ATA​GCA​GG	TGT​TCA​AAC​TTC​TGC​TCC​TGA	Albermann et al. (2005)
*MRP1*/ABCC1	NM_004996.3 GI: 134142336	ATG​TCA​CGT​GGA​ATA​CCA​GC	GAA​GAC​TGA​ACT​CCC​TTC​CT	Gutmann et al. (1999)
*BCRP*/ABCG2	NM_004827.2 GI: 62526032	AGA​TGG​GTT​TCC​AAG​CGT​TCA​T	CCA​GTC​CCA​GTA​CGA​CTG​TGA​CA	Sauerbrey et al. (2002)
β2mg	X07621.1 GI: 29298	CCA​GCA​GAG​AAT​GGA​AAG​TC	CAT​GTC​TCG​ATC​CCA​CTT​AAC	Oselin et al. (2003)

### 2.7 Western blot

Total proteins were extracted from each cell pellet utilizing 100 μL of RIPA lysis buffer with protease inhibitors (Thermo Fisher Scientific), and total protein concentrations were quantified using the BCA protein assay kit (Thermo Fisher Scientific). Cell lysate samples, each containing 50 μg of total protein, were applied to gradient 4%–20% Mini-PROTEAN^®^ TGX Stain-Free™ SDS-PAGE gels (Bio-Rad Laboratories Inc.; CA, United States). The resolved proteins were subsequently transferred to 0.2 Trans-Blot^®^ Turbo™ PVDF membranes utilizing a Trans-Blot^®^ Turbo™ Transfer System (Bio-Rad Laboratories Inc.). Membranes were incubated for 15 min with SuperBlock™ T20 buffer (TBS-T; Thermo Fisher Scientific) and subsequently exposed to primary antibodies (1:1,000 dilution for all antibodies) overnight at 4 °C. Thereafter, the membranes were rinsed with TBS-T and incubated for 1 h with WestVision™ secondary anti-mouse or anti-rabbit IgG antibodies (1:10,000) conjugated to peroxidase micropolymer (Vector Laboratories Inc., CA, United States). The membranes underwent washing, and the signals were developed using the SignalFire™ Plus ECL Reagent (Cell Signaling Technology Inc.). The images were obtained using the ChemiDocTM XRS + imaging system (BioRad Laboratories Inc.), and the density of each protein band was quantified and normalized against the densitometry of the GAPDH band using ImageJ software (https://imagej.nih.gov/ij/), as previously described.

### 2.8 Statistical analysis

The data is presented as the mean value plus or minus the standard deviation. The IC_50_ value was calculated by analyzing dose-response curves to identify the concentration of tested substances that inhibit half % of the cell growth under the assay conditions. The dose-response curves were computed using a four-parameter logistic curve using GraphPad Prism^®^ software (Version 8, San Diego, CA, United States). The sets of data were analyzed using a one-way analysis of variance to determine any differences at *p*-value <0.05. Post Hoc tests with ANOVA using Tukey’s Test was applied to determine the significant difference of every possible pairwise comparison.

## 3 Results

### 3.1 Cytotoxicity

**FIGURE 2:**
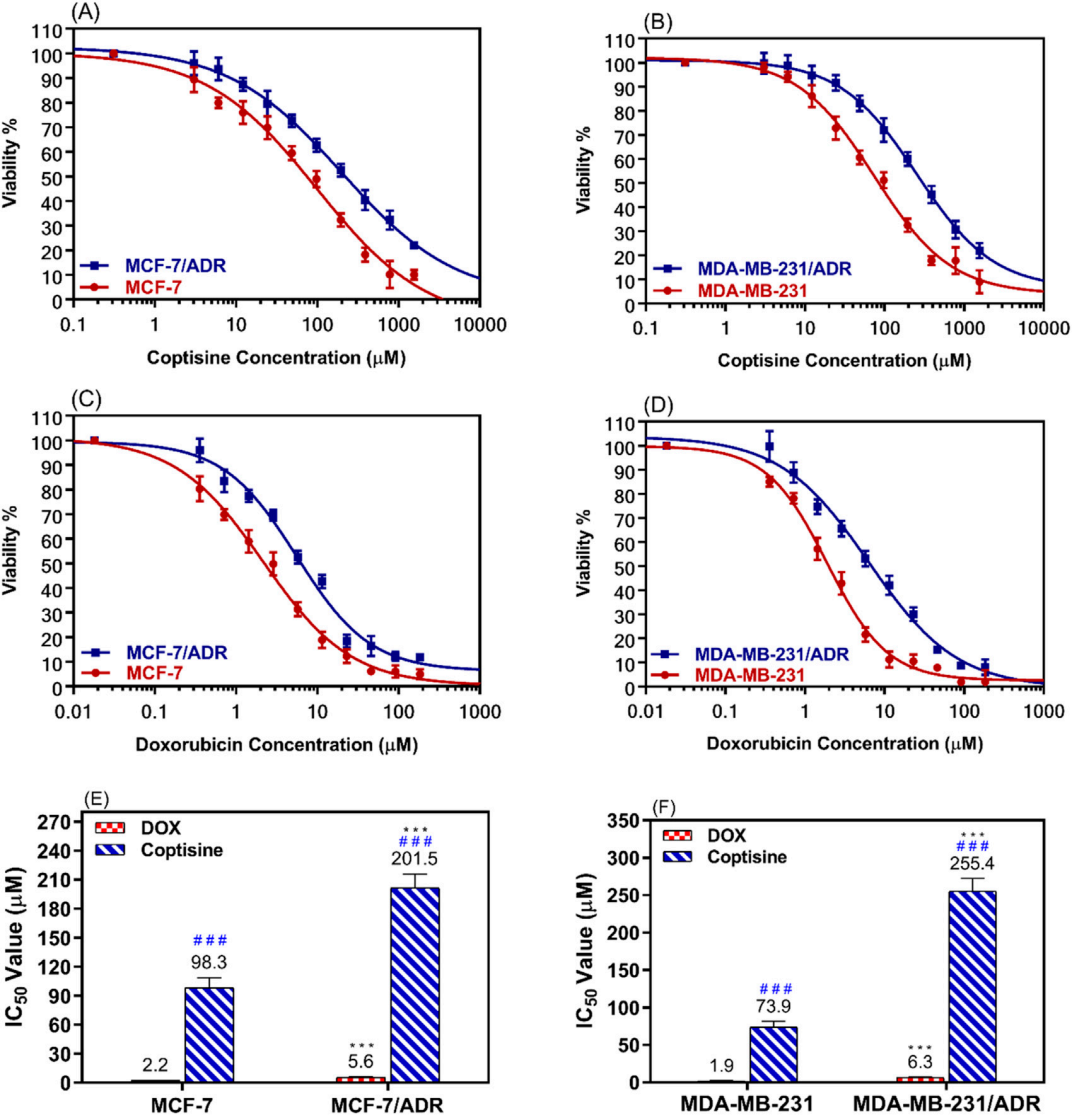
The cytotoxic effects of coptisine and doxorubicin on both sensitive and resistant breast cancer cell lines. The dose-response curve (DRC) of coptisine (COP) 0.3–1,561 μM against MCF-7 and MCF-7/ADR **(A)** against MDA-MB-231 and MDA-MB-231/ADR **(B)** DRC of doxorubicin (DOX) (0.2–184 μM) against MCF-7 and MCF-7/ADR **(C)** against MDA-MB-231 and MDA-MB-231/ADR **(D)** and the IC_50_ values of COP **(E)** and DOX **(F)** against the tested cell lines. *** = significant difference (P< 0.001) compared to sensitive cells, while ### = significant difference (P< 0.001) compared to DOX values.

The dose-response curve for various doses of COP and DOX was determined after incubating resistant cells (MCF-7/ADR and MDA-MB-231/ADR) and parent cells (MCF-7 and MDA-MB-231) for 24 h (Figures 2A, B). The results were compared to those obtained with DOX alone (Figures 2C, D).

The MCF-7/ADR cells exhibited approximately a two-fold increase in resistance to COP cytotoxic effects compared to their parent cells, as seen in Figure 2E. Similarly, the MDA-MB-231/ADR cells demonstrated a 3.5-fold increase in resistance, as depicted in Figure 2F. Both cell types exhibited a cytotoxic pattern when treated with DOX, with a 2.6-fold and 3.3-fold increase in cytotoxicity compared to parent cells, respectively. The IC_50_ values of COP in MCF-7 and MCF-7/ADR cells were 98.3 ± 10.3 μM and 201.5 ± 14.4 μM, respectively. DOX exhibited a more cytotoxic effect against MCF-7 and MCF-7/ADR cells at 2.2 ± 0.18 μM and 5.6 ± 0.49 μM, respectively (Figure 2E). Regarding the MDA-MB-231 and MDA-MB-231/ADR, the IC_50_ values of COP were 73.9 ± 7.5 μM and 255.4 ± 16.9 μM, while the IC_50_ of DOX were 1.9 ± 0.17 μM and 6.3 ± 0.73 μM, respectively (Figure 2F).

### 3.2 Enhance the DOX cytotoxicity by COP combination

**FIGURE 3:**
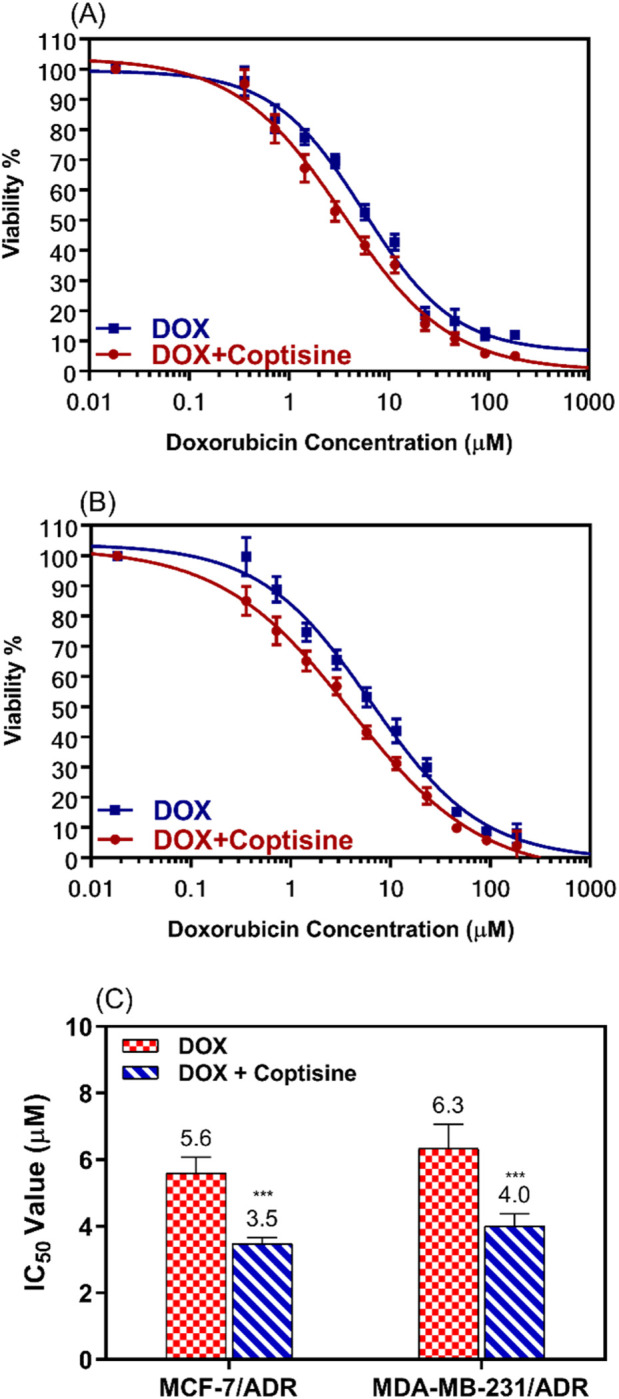
The cytotoxic effects of doxorubicin in combination with 31 μM of coptisine on both resistant breast cancer cell lines. The dose-response curve (DRC) for the drug combinations doxorubicin with coptisine (DOX + COP) against MCF-7/ADR **(A)**, against MDA-MB-231/ADR **(B)**, and the IC_50_ values of doxorubicin (DOX) with and without coptisine (COP) **(C)** were determined in the tested cell lines. *** = a significant difference of DOX + COP (P< 0.001) compared to DOX alone.

Based on the cytotoxicity data showed in Figure 2, 31 μM (10 μg/mL) of COP was selected to use that concentration because still not cytotoxic alone on both parental and resistant cell models (>80% cell viability). The ability of COP to reverse the DOX resistance in the resistance breast cells was evaluated using the cell proliferation assay. MCF-7/ADR and MDA-MB-231/ADR were incubated with several doses of DOX with or without COP for 24 h (Figures 3A, B).

The cytotoxicity of DOX was enhanced by combination with COP as observed from the dose-response curve in both cell lines.

IC_50_ values of DOX were significantly decreased 1.6-fold in both cell lines; from 5.6 ± 0.5 μM to 3.5 ± 0.18 μM (P< 0.001) in MCF-7/ADR and from 6.3 ± 0.73 μM to 4.0 ± 0.37 μM (P< 0.001) in MDA-MB-231/ADR (Figure 3C).

### 3.3 Synergism interaction of DOX with COP

**FIGURE 4:**
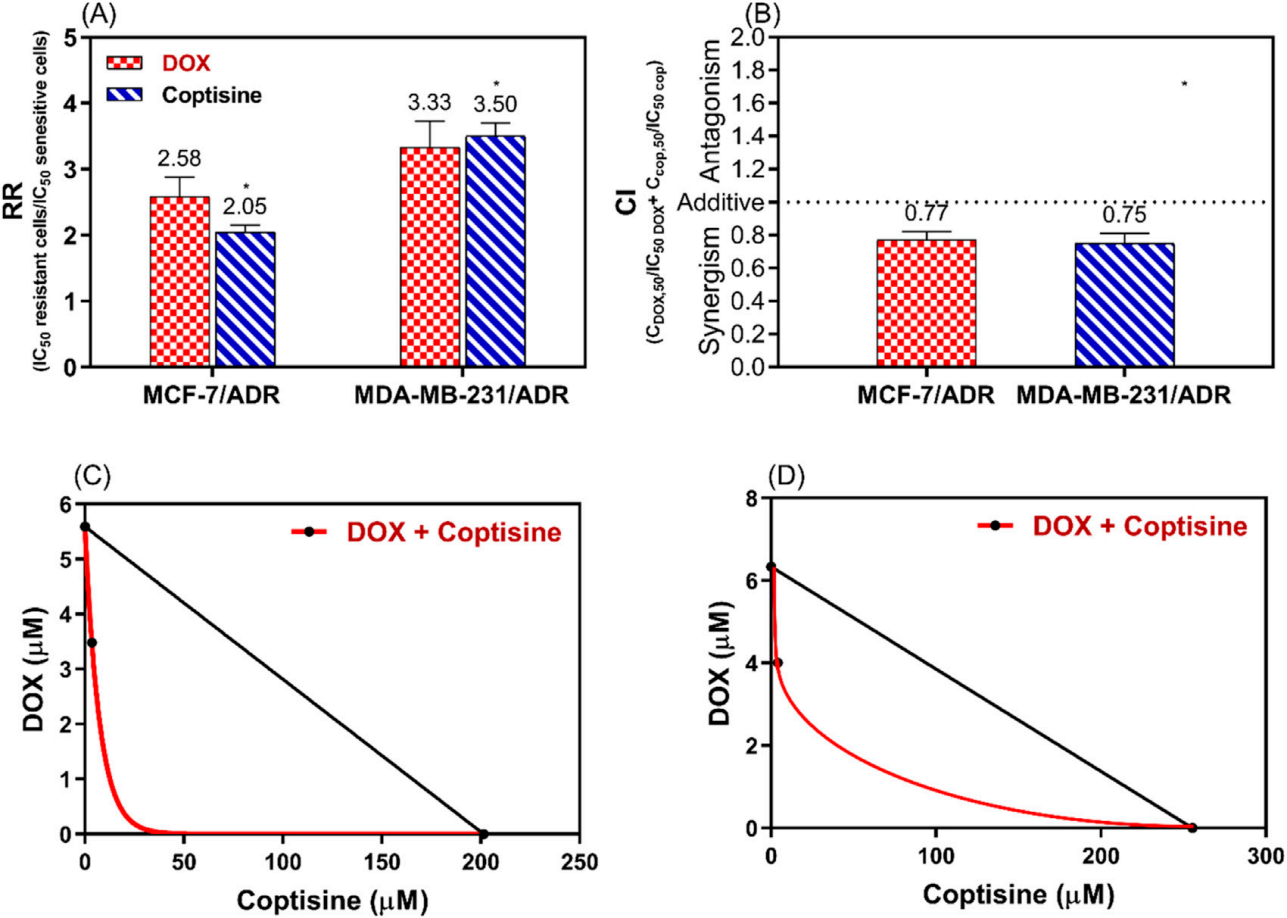
The analysis of the synergistic interaction between DOX and COP in MCF-7/ADR and MDA-MB-231/ADR. This analysis is conducted using the resistance ratio (RR) in **(A)**, the combination index (CI) in **(B)**, and the isobologram (IB) in **(C, D)** of both cell lines, respectively. * = significant difference with a *p* < 0.05 for the RR of COP compared to DOX.

To evaluate the synergistic interaction between DOX and COP the resistance ratio (RR), the combination index (CI), and the isobologram (IB) were used (Figure 4). The RR of COP was significantly different (2.05 and 3.5) than DOX (2.58 and 3.33) P< 0.05 for MCF-7/ADR and MDA-MB-231/ADR, respectively (Figure 4A). To confirm the synergistic interaction CI was 0.77 and 0.75 (<1) for the DOX + COP combination in both cells, respectively (Figure 4B) and IB confirm the synergism (Figure 4C, D).

The DRI of COP was 6.5 and 8.2 and DRI of DOX was 1.6 and 1.57 for MCF-7/ADR and MDA-MB-231/ADR, respectively.

### 3.4 The modulation of ABC-Transporter functions

**FIGURE 5:**
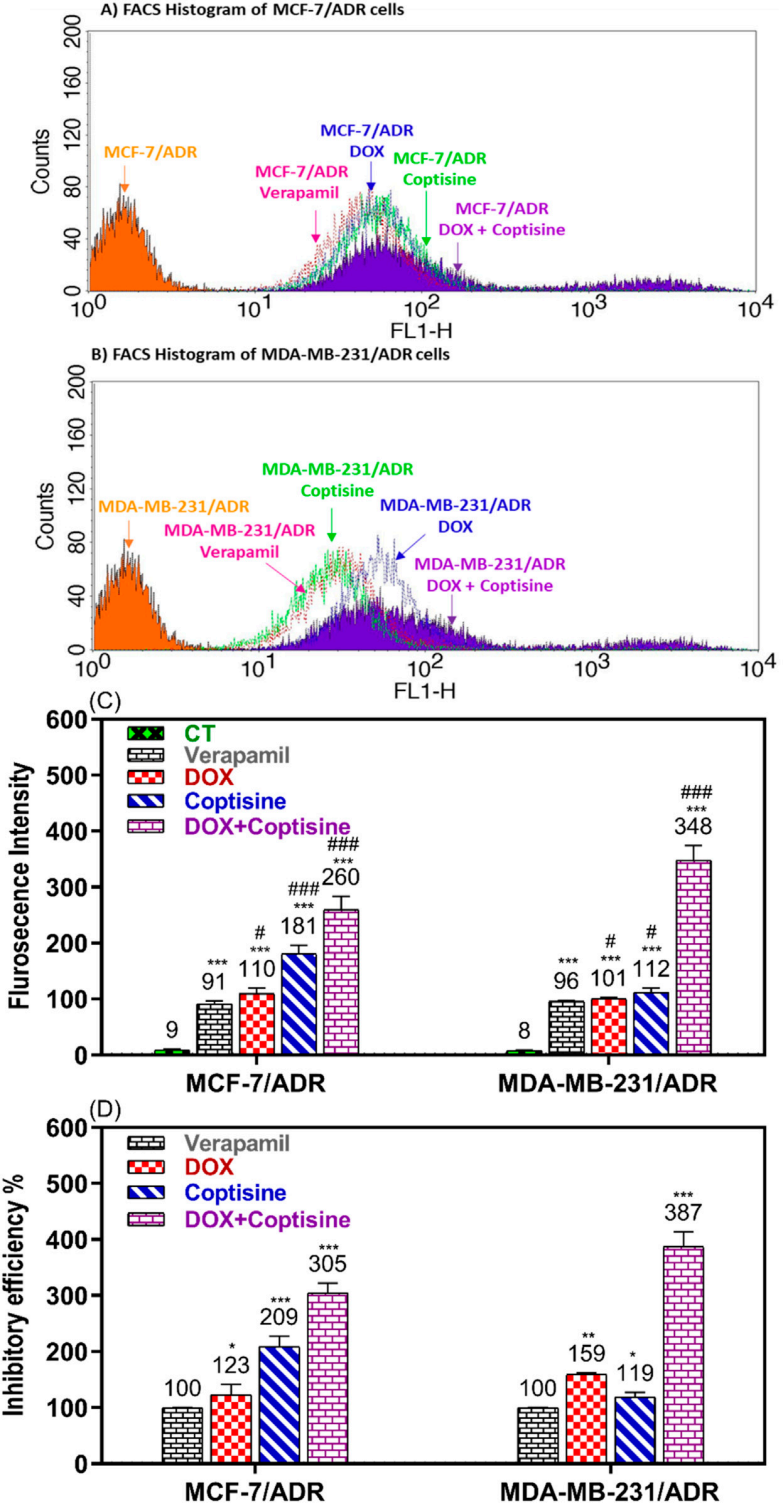
Flow cytometry ABCTs functional assay. The flow cytometry histogram of Rho123 accumulation fluorescence intensity in **(A)** MCF-7/ADR and **(B)** in MDA-MB-231/ADR after doxorubicin (DOX), coptisine (COP), and their combination treatment compared to untreated and verapamil (VER) treated cells. The fluorescence intensity (FI) shift from left to right on the histogram indicated increased Rho123 accumulation and ABCT inhibition. **(C)** The flurosecence intenstity of histogram statatitical data. **(D)** The inhibitory efficiency % (IE%) was calculated by comparing the tested sample’s FI to untreated and VER-treated cells. *, **, and *** indicated the levels of the significant difference of IE% P< 0.05, P< 0.01, and P< 0.001 compared to VER-treated cells, respectively.

Rhodamine 123 is a fluorescence dye and substrate of ABCTs mainly P-gp/MDR1 and MRP1 was used to evaluate the ability of COP to modulate the ABCT function using FACS (Figure 5). In the FACS histogram, the fluorescence intensity of Rho123 was shifted from left to right after treatment with COP, DOX, and DOX + COP indicating the increase in the accumulation of Rho123 by treatment comparing to VER in reistant cells (Figures 5A, B). The mean of fluroscence intensity of Rho123 accumulation of untreated cells 9.3 and 8 and significant increased by tratemnt with COP and DOX-COP compared with VER in MCF-7/ADR and MDA-MB231/ADR, respectively (Figure 5C). The fluorescence intensity % (FI) was calculated to compare the modulatory effect of each sample to the positive control; VER (100%). The FI was increased by the treatment with DOX to 1.23 and 1.59-fold and with COP to 2.1 and 1.2-fold of treatment with VER (1). The COP combination with DOX significantly improved the FI to 3.1 and 3.9-fold of treatment with VER (P< 0.001) Figure 5D.

### 3.5 The ABCTs gene expression

**FIGURE 6:**
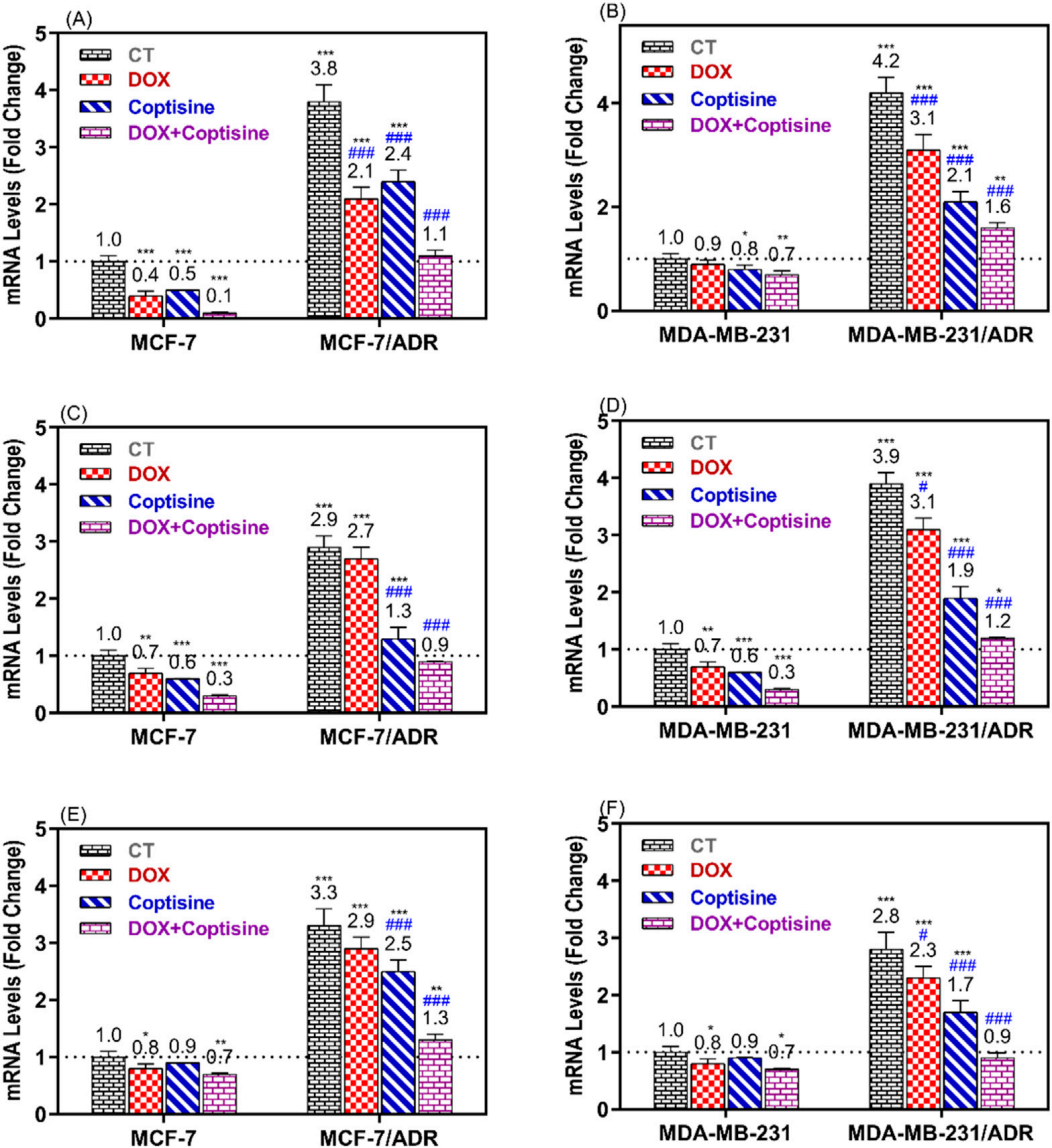
RT-PCR was used to compare the expression of ABCTs genes in MCF-7/ADR and MDA-MB-231/ADR cells treated with DOX 1.8 μM, COP 31 μM, and their combination to that of untreated cells. The gene expression of MDR1 **(A, B)**, BCRP **(C, D)**, and MRP1 **(E, F)** in MCF-7/ADR and MDA-MB-231/ADR cells in comparison to their parent cells, respectively. There is a significant fold change in gene expression when compared to the untreated parent cells (1). *, **, and *** indicated the levels of the significant difference of gene expressions P< 0.05, P< 0.01, and P< 0.001 compared to their expression in the parent untreated cells, while # and ### indicated the levels of the significant difference of gene expressions P< 0.05 and P< 0.001 compared to their expression in the resistant untreated cells, respectively.

The expressions of ABCTs genes related to MDR were determined in resistant and sensitive cell lines after treatment with COP, DOX, and DOX + COP using RTPCR (Figure 6). The expressions of *P-gp/MDR1, BCRP, and MRP1* genes were significantly upregulated in resistant cell MCF-7/ADR (3.8, 2.9, and 3.3-folds, P< 0.001) and MDA-MB-231/ADR (4.2, 3.9, and 2.8-folds, P< 0.001) compared to sensitive parent cells. The treatment with DOX and COP resulted in the significant downregulation of the ABCT gene in sensitive and resistant cells. The combinations of DOX with COP significantly decreased the expression of ABCT gens mainly to ∼1 fold of resistant cells indicating the reverse of their DOX-resistance.

### 3.6 The ABCTs protein expression

**FIGURE 7:**
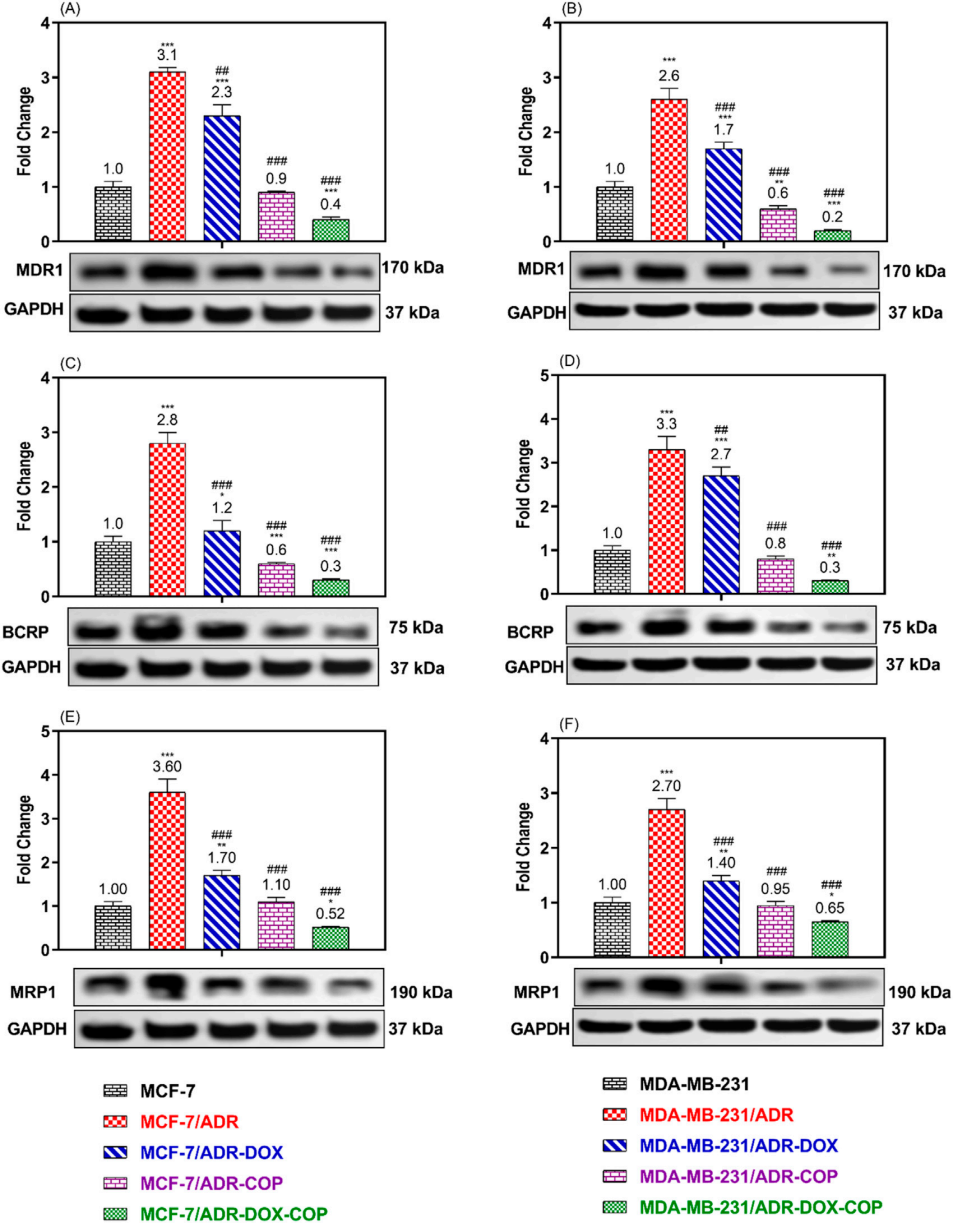
WB was used to compare the expression of ABCTs protein in MCF-7/ADR and MDA-MB-231/ADR cells treated with DOX 1.8 μM, COP 31 μM, and their combination to that of untreated cells and senstive parent cells. The Protein expression of P-gp/MDR1 **(A, B)**, BCRP **(C, D)**, and MRP1 **(E, F)** in MCF-7/ADR and MDA-MB-231/ADR cells in comparison to their parent cells, respectively. There is a significant fold change in gene expression when compared to the untreated parent cells (1). *, **, and *** indicated the levels of the significant difference of protein expressions P< 0.05, P< 0.01, and P< 0.001 compared to their expression in the parent untreated cells, while # and ### indicated the levels of the significant difference of protein expressions P< 0.05 and P< 0.001 compared to their expression in the resistant untreated cells, respectively.

To verify the modulatory impact of COP and its combination with DOX on MDR cells. The expressions of ABCT proteins associated with MDR were assessed in resistant and sensitive cell lines following treatment with COP, DOX, and DOX + COP using Western blotting (Figure 7). The expressions of P-glycoprotein/MDR1, BCRP, and MRP1 proteins were markedly upregulated in resistant cell lines MCF-7/ADR (3.1, 2.8, and 3.6-folds, P< 0.001) and MDA-MB-231/ADR (2.6, 3.3, and 2.7-folds, P< 0.001) in comparison to their sensitive parental cells. The use of DOX and COP led to a substantial downregulation of the ABCT proteins in both resistant cells. The administration of COP and its combinations with DOX markedly reduced the expression of ABCT protein to less than ∼ 1-fold in resistant cells, indicating a reversal of DOX resistance.

## 4 Discussion

The occurrence of MDR during chemotherapy is a significant factor that limits clinical treatment and results in chemotherapy ineffectiveness. P-gp/MDR1, BCRP, and MRP1 are members of the ABC transporter family. They can interact with chemotherapeutic medicines such as DOX and expel intracellular pharmaceuticals to extracellular cells through ATP hydrolysis, causing MDR. Studies have demonstrated the efficacy of Traditional Chinese Medicine (TCM) as a supplementary medication for chemotherapy in counteracting MDR. TCM achieves this by reducing the function and expression of ABC transporters in cancer cells. BC patients have a favorable outcome and can achieve a complete recovery through timely and suitable medical intervention during the initial stages of the illness (Waks et al., 2016). The primary cause of death from BC is MDR, which can lead to metastatic disease. The main obstacle to finding a cure for the disease is the spread of cancer throughout the body. Therefore, the reverse of MDR is an important goal in the BC investigation. Because of this, there has been significant interest in a new medicine or phytochemical product that could inhibit MDR in breast cancer (El-Readi et al., 2024). Particularly, compounds that have minimal adverse effects have attracted attention (Eslami et al., 2024). For this study, MCF7 and MDA-MB-231 cells were used as parent cells to demonstrate a typical scenario. Despite being both invasive ductal/BC cells, they exhibit numerous phenotypic/genotypic distinctions. MCF7 cells depend on hormones, showing positive expression of estrogen, progesterone, and endothelial growth factor receptors (ER, PR, and EGF). MDA-MB-231 cells, on the other hand, are known for being triple-negative (Theodossiou et al., 2019). The lack of estrogen receptor (ER) in MDA-MB-231 has made it unresponsive to antiestrogen therapy, including the commonly used selective estrogen receptor modulator tamoxifen in BC prevention and treatment (Radmacher and Simon, 2000). MCF7 cells are primarily based on oxidative phosphorylation for ATP production under normal oxygen conditions but increase their glycolytic activity when oxygen is limited. In contrast, MDA-MB-231 cells are predominantly based on glycolysis for ATP production regardless of oxygen availability, indicating a Warburg-type metabolism (Theodossiou et al., 2019). While MDA-MB-231 cells show a more mesenchymal phenotype and have been found to have MDR, MCF7 cells have an epithelial phenotype (Theodossiou et al., 2019). To examine the effect of COP on sensitive and resistant cell lines, this study created DOX-resistant cell lines as *in vitro* models of acquired MDR through continuous treatment with a low dosage of DOX (1.8 μM). This model was successful because resistant cell lines had strong expressions of the ABCTs genes and proteins. The study aimed to investigate the effects of COP on MDR in various BC cell lines and their commonly used resistant models. It also examined the synergistic interaction between COP and DOX in modulating ABCTs in resistant BC.

The study found that COP had a moderate cytotoxic effect on the tested cell lines, although this effect was not as strong as the cytotoxic medication DOX. However, the combination of non-toxic concentration of COP 31 μM (10 μg/mL) and DOX significantly enhanced the cytotoxicity of DOX in the resistant cell lines. Figures 2, 3 demonstrate this synergy in both resistant and sensitive breast cancer cell lines. The results suggest that COP could serve as an adjuvant drug in the treatment of BC. Thus, the combination of DOX demonstrates a potent cytotoxic impact at low dosages with few adverse effects, particularly in drug-resistant BC.

Furthermore, the COP inhibited ABCT’s function and expression, as evidenced by a notable increase in Rho123 accumulation (Figure 5) and downregulation of ABCT gene (Figure 6) and protein expression (Figure 7). Both DOX and VER increased Rho123 accumulation, indicating ABCT inhibition at the dose used. DOX and P-gp interaction depends on cancer type, DOX dose, and cancer cell P-gp expression. DOX can increase chemotherapy sensitivity by inhibiting P-gp. In other cases, it may not overcome MDR. Normally a substrate for ABCT transporters, DOX can also inhibit them under certain conditions. To overcome drug resistance, optimizing its use in cancer treatment requires understanding this dual role (Robey et al., 2018). Research has demonstrated that doxorubicin inhibits ABC transporter efflux activity at concentration-dependent rates. At high doses, it can bind to P-gp and other transporters, making them less effective at effluxing other substrates. The drug concentration and cellular context are major factors in this dual role. This dose-dependent inhibition lends credence to the idea that doxorubicin can play a dual role as substrate and inhibitor, depending on the concentration and saturation of the transporter. DOX is effectively refluxed by ABC transporters at therapeutic doses typically used in clinical settings, resulting in diminished efficacy. On the other hand, when doxorubicin concentrations are increased in an experimental setting, the transporters become saturated, and their efflux function is inhibited (Gottesman et al., 2016).

**FIGURE 8:**
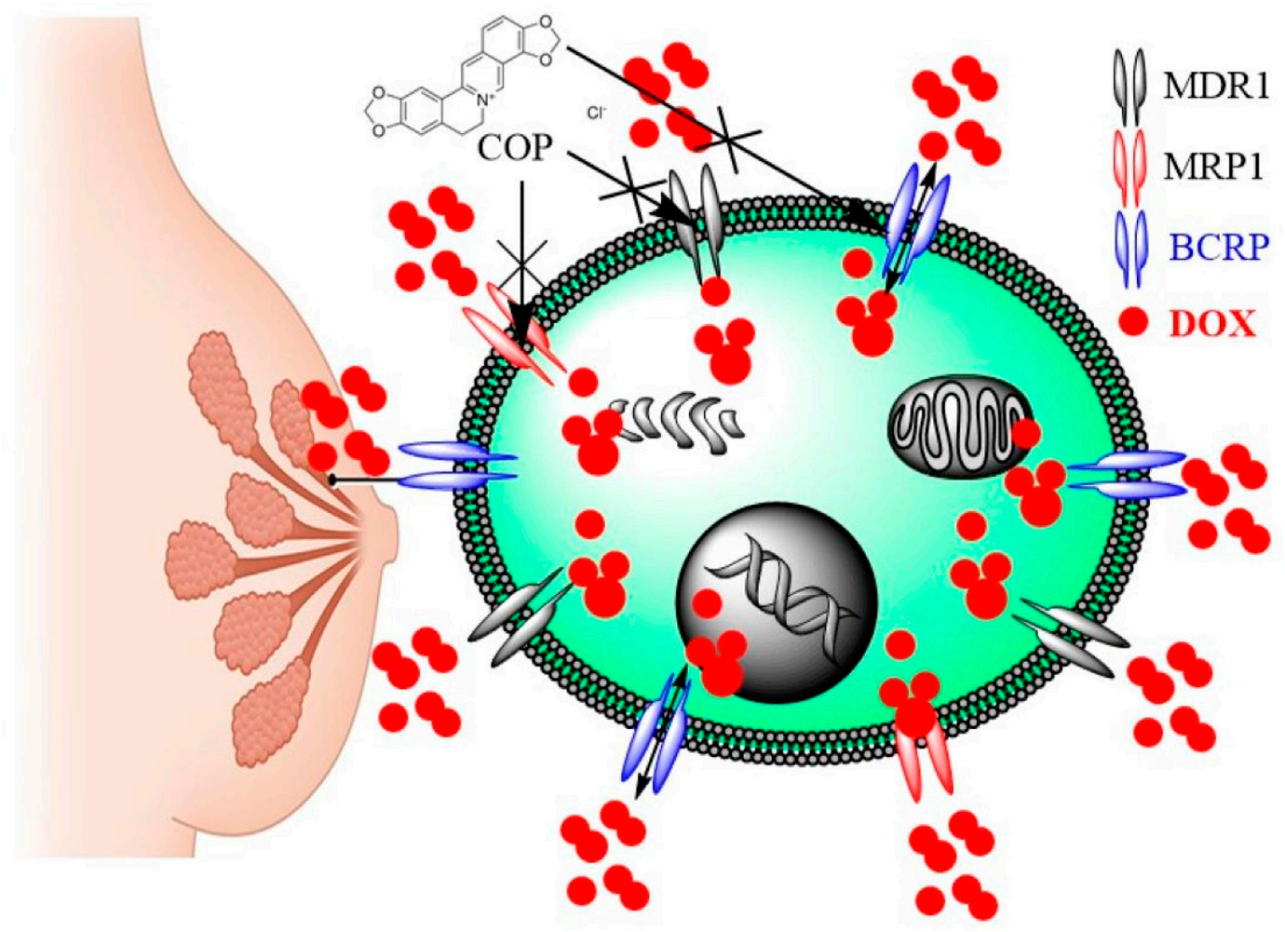
The MDR reversal mechanisms of COP in MDR breast cancer cells.

The data indicated that COP effectively reversed multidrug resistance (MDR) in BC and restored the sensitivity of DOX-resistant cells to its cytotoxic effects. Previously, COP reduced MDA-MB-231 adhesion, migration, and invasion *in vitro* by downregulating MMP-9 and increasing TIMP-1, presumably due to its anti-metastatic effect on BC (Li et al., 2014). Previously, IQA demonstrated concentration-dependent inhibition of P-gp/MDR1 activity in both Caco-2 and CEM/ADR5000 cells, effectively reversing their resistance to doxorubicin (El-Readi et al., 2013). The expression analysis revealed a common set of key genes associated with apoptosis, cell cycle, and drug metabolism. Exposure of Caco-2 cells to isoquinoline alkaloid (IQA) led to a notable reduction in mRNA levels of MDR1, MRP1, BCRP, CYP3A4, GST, and hPXR (El-Readi et al., 2013). The postulated mechanism for the chemosensitizing effect of IQA involves the modification of the ATPase activity of MDR1 (Ativui et al., 2021). Understanding the mechanism by which IQA reduces MDR in BC is important. In line with the current findings, researchers have used the MTT assay to assess the drug resistance and cytotoxicity of berberine (Ber) and doxorubicin (DOX), either separately or in combination, in MCF-7/DOX^Fluc^, which stably expresses the firefly luciferase reporter gene. Ber had a synergistic impact in enhancing the inhibitory effect of DOX on cell growth. *In vivo*, Ber significantly decreased the release of d-luciferin potassium salt in the MCF-7/DOX^Fluc^ cell line, suppressing the activity of P-gp/MDR1 and MRP1 in these cells. Additionally, it enhanced the uptake of DOX in tumor tissues by reversing multidrug resistance (MDR) by inhibiting the efflux of ABCTs and reducing their expression (Qian et al., 2021). Figure 8 summarizes the MDR reversal mechanism of COP that boosted DOX concentration in resistant cells and improved its cytotoxicity in MCF-7/ADR and MDB-MB-231/ADR cells, and that it could overcome MDR by inhibiting ABCT function and expression in a resistant BC.

## 5 Conclusion

This study demonstrated that COP could overcome MDR by inhibiting ABCT function and expression in a resistant BC. COP increases DOX concentration in resistant cells and enhances its cytotoxicity in MCF-7/ADR and MDB-MB-231/ADR cells. More *in vitro* studies are required to validate the effect of COP on ABCT targets of ABCTs, such as ATPase activities and drug accumulation studies. *In vivo* studies are needed to make sure that the current results are correct and to see how well COP and DOX combinations work to reverse multidrug resistance (MDR) in breast cancer models in animals.

## Data Availability

The original contributions presented in the study are included in the article/Supplementary Material, further inquiries can be directed to the corresponding author.
