# Supplementary material for: Coptisine enhances the sensitivity of chemoresistant breast cancer cells by inhibiting the function and expression of ABC transporters
Source: Front Pharmacol. 2024 Dec 3;15:1472458. doi: 10.3389/fphar.2024.1472458 (PMC11650208; doi:10.3389/fphar.2024.1472458)

Detection of MDR1 protein (170 KD) by WB

1 = MCF-7
2 = MCF-7/ADR
3 = MCF-7/ADR-DOX
4 = MCF-7/ADR-COP
5 = MCF-7/ADR-DOX-COP

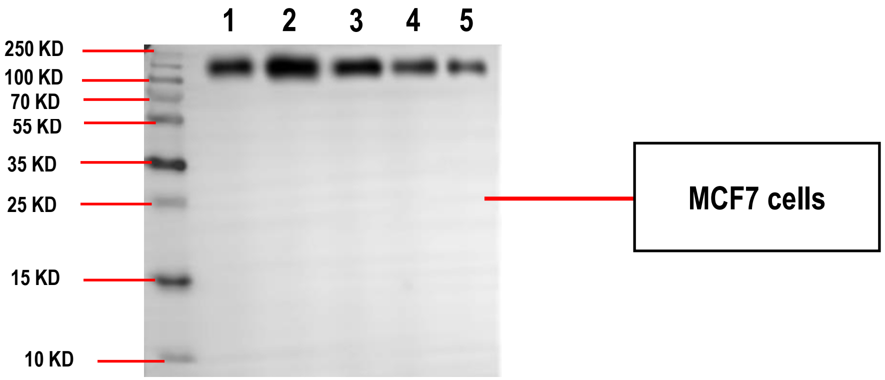

1 = MDA-MB-231
2 = MDA-MB-231/ADR
3 = MDA-MB-231/ADR-DOX
4 = MDA-MB-231/ADR-COP
5 = MDA-MB-231/ADR-DOX-COP

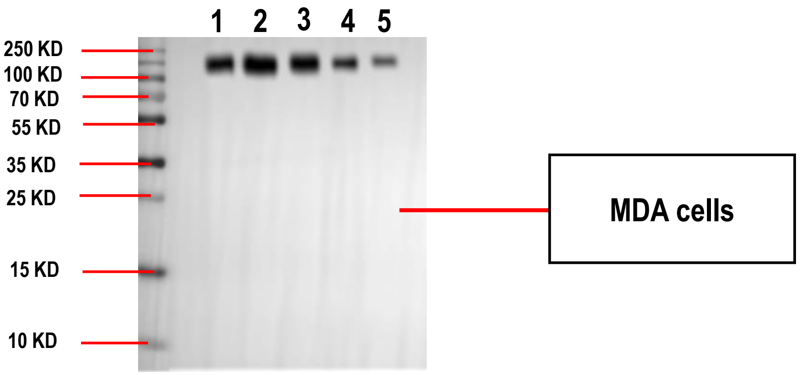

Detection of BCRP protein (75 KD) by WB

1 = MCF-7
2 = MCF-7/ADR
3 = MCF-7/ADR-DOX
4 = MCF-7/ADR-COP
5 = MCF-7/ADR-DOX-COP

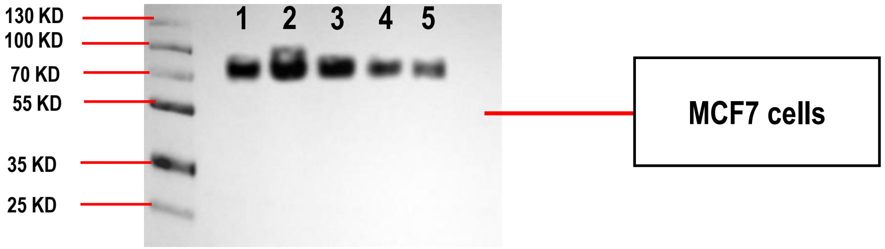

1 = MDA-MB-231
2 = MDA-MB-231/ADR
3 = MDA-MB-231/ADR-DOX
4 = MDA-MB-231/ADR-COP
5 = MDA-MB-231/ADR-DOX-COP

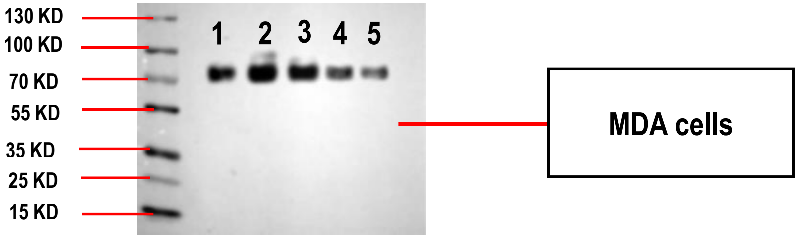

Detection of MRP1 protein (190 KD) by WB

1 = MCF-7
2 = MCF-7/ADR
3 = MCF-7/ADR-DOX
4 = MCF-7/ADR-COP
5 = MCF-7/ADR-DOX-COP

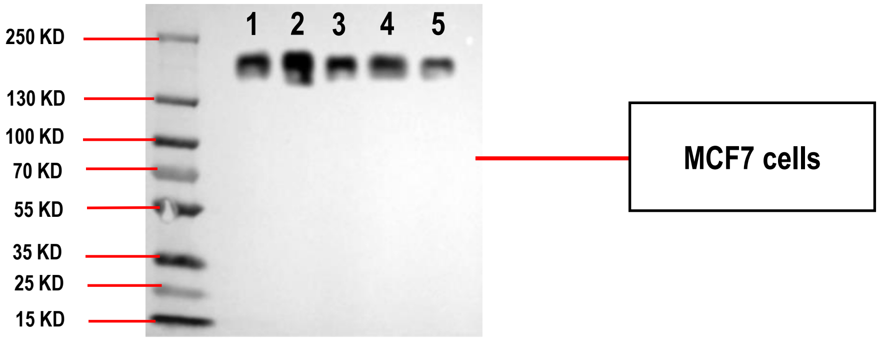

1 = MDA-MB-231
2 = MDA-MB-231/ADR
3 = MDA-MB-231/ADR-DOX
4 = MDA-MB-231/ADR-COP
5 = MDA-MB-231/ADR-DOX-COP

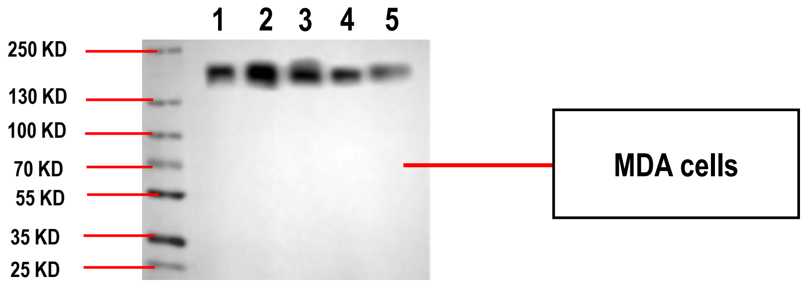

Detection of GAPDH protein (37 KD) by WB

1 = MCF-7
2 = MCF-7/ADR
3 = MCF-7/ADR-DOX
4 = MCF-7/ADR-COP
5 = MCF-7/ADR-DOX-COP

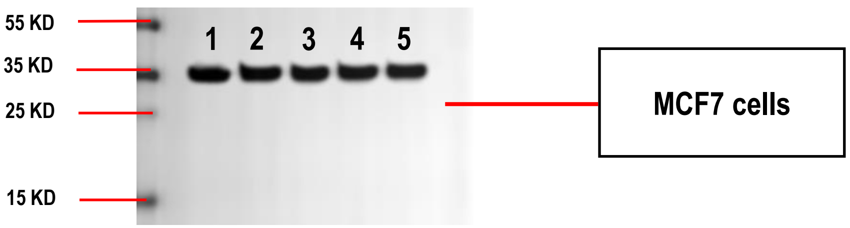

1 = MDA-MB-231
2 = MDA-MB-231/ADR
3 = MDA-MB-231/ADR-DOX
4 = MDA-MB-231/ADR-COP
5 = MDA-MB-231/ADR-DOX-COP

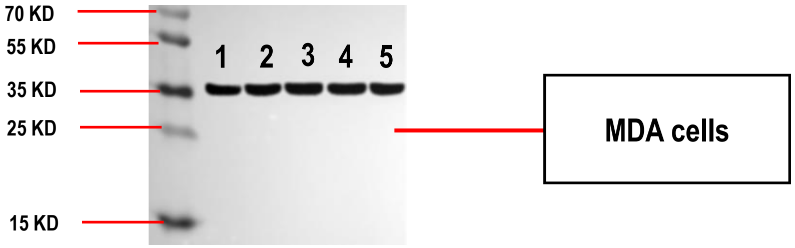

Supplement: Supplementary file 1 [file DataSheet1.PDF]
